# P-56. Respiratory Syncytial Virus (RSV) Vaccine Uptake among Adults ≥60 years old in a Large Integrated Healthcare System in the U.S

**DOI:** 10.1093/ofid/ofae631.263

**Published:** 2025-01-29

**Authors:** Sara Y Tartof, Bradley Ackerson, Banshri Kapadia, Vennis Hong, Sally Shaw, Brigitte Spence, Jeff Slezak, Julie Stern, Gregory Davis, Gabriella Goodwin, Hung Fu Tseng, Parag Mahale

**Affiliations:** Kaiser Permanente Southern California, Pasadena, CA; Kaiser Permanente Southern California, Pasadena, CA; Kaiser Permanente, Southern California, Pasadena, California; Kaiser Permanente, El Monte, California; Kaiser Permanente Southern California, Pasadena, CA; Kaiser Permanente Southern California, Pasadena, CA; Kaiser Permanente Southern California, Pasadena, CA; Kaiser Permanente Southern California, Pasadena, CA; Kaiser Permanente, Southern California, Pasadena, California; Kaiser Permanente, Southern California, Pasadena, California; Kaiser Permanente Southern California, Pasadena, CA; Kaiser Permanente Southern California, Pasadena, CA

## Abstract

**Background:**

In 2023, the FDA approved two new vaccines to protect adults ≥ 60 years old from severe RSV disease. CDC’s Advisory Committee on Immunization Practices recommended a shared decision-making approach between age-eligible adults and their providers for a single dose of either RSVPreF3 (Arexvy, GSK) or RSVpreF (AbrysvoTM, Pfizer) vaccines. We examined uptake among age-eligible Kaiser Permanente Southern California (KPSC) members to assess demographic and socio-economic disparities while accounting for clinical characteristics.

**Figure:**
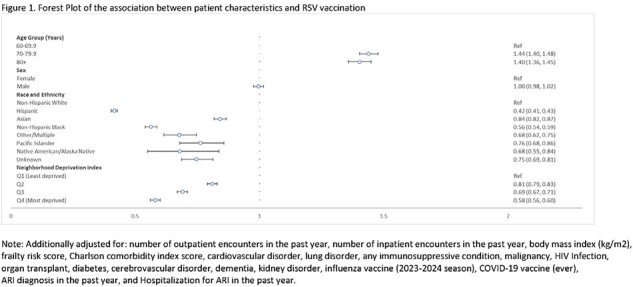

**Methods:**

The cohort included KPSC members ≥ 60 years old with ≥ 1 year of KPSC membership on September 23, 2023 (i.e., study start and date of first KPSC RSV vaccination). Follow-up ended on either December 31, end of KPSC membership, or death. RSV vaccination included either RSVPreF3 or RSVpreF vaccination. Logistic regression models estimated adjusted odds ratios (OR) and 95% confidence intervals (CI) for factors associated with uptake, including demographics and neighborhood deprivation index (NDI) quartiles, prior healthcare utilization, comorbidities, prior acute respiratory infection (ARI) diagnosis and ARI hospitalization, and prior COVID-19 and influenza vaccinations.

Table 1 (page 1)

**Figure:**
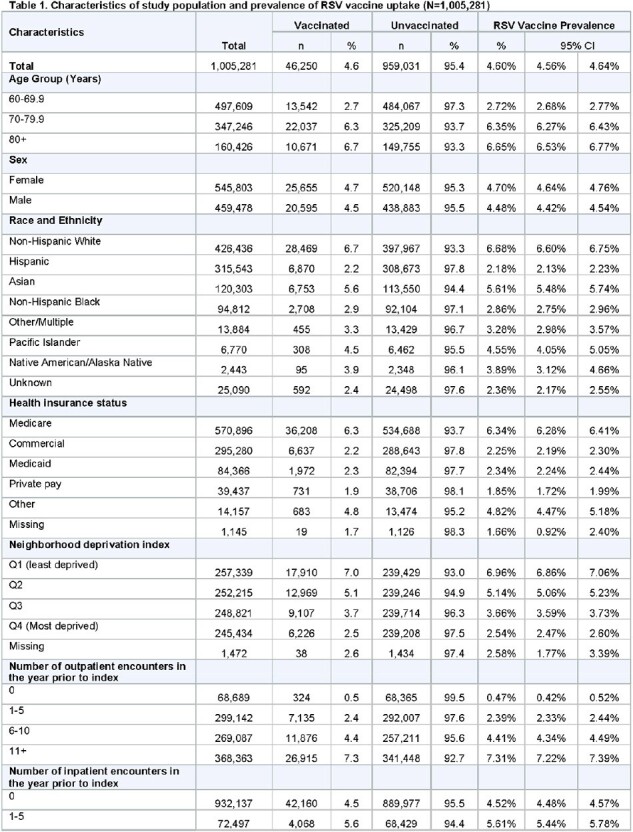

**Results:**

Among 1,005,281 KPSC members who met the inclusion criteria, the median age was 70 years (IQR: 64–76), 54.3% were female, 42.4% were non-Hispanic White, 31.4% were Hispanic, 12.0% were Asian, and 9.4% non-Hispanic Black. Overall, 4.6% were RSV vaccinated. Compared to those < 70 years, older members had increased odds of vaccination (70-79 years: OR = 1.44 [95% CI: 1.40, 1.48]; 80+ years: 1.40 [1.36, 1.45]). Hispanic (0.42 [0.41, 0.43]), Asian (0.84 [0.82, 0.87]), and non-Hispanic Black (0.56 [0.54, 0.87]) members had lower odds of vaccination compared to non-Hispanic White members. Similarly, members in the highest NDI quartile (i.e., most deprived) had a lower odds of vaccination (0.58 [0.56, 0.60]) compared to those in the lowest quartile.

Table 1 (page 2)

**Figure:**
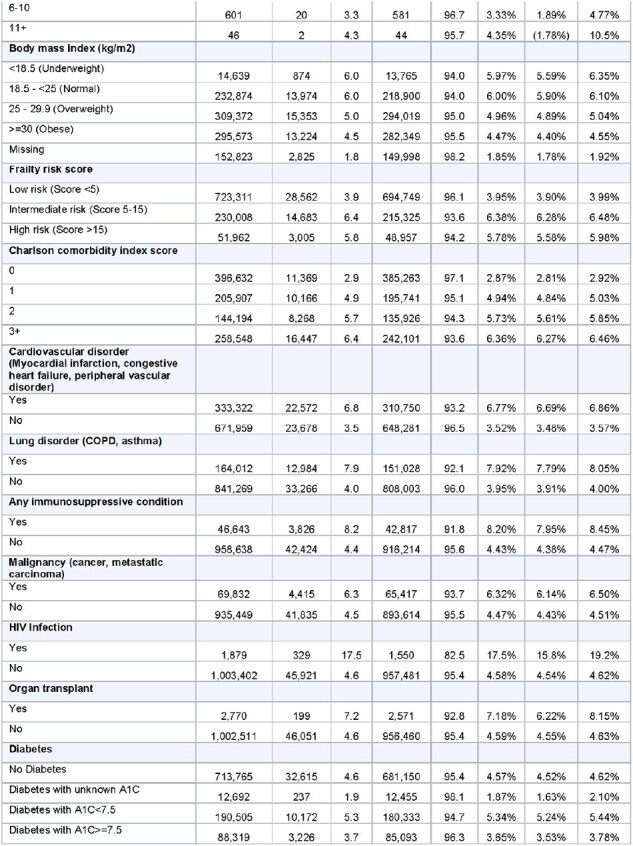

**Conclusion:**

RSV vaccination among KPSC members was low in the first 3 months of implementation and reflected racial and socio-economic disparities in uptake, even when accounting for clinical characteristics. Targeted strategies in vulnerable populations are needed to maximize the impact of RSV vaccination.

Table 1 (page 3)

**Figure:**
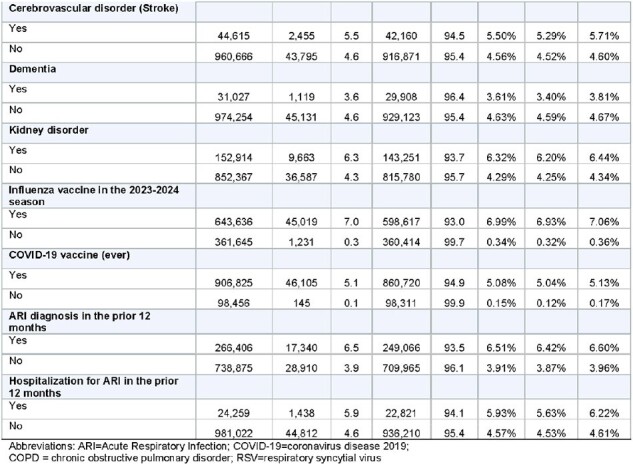

**Disclosures:**

**Sara Y. Tartof, PhD MPH**, GSK: Grant/Research Support|Pfizer Inc: Grant/Research Support **Bradley Ackerson, MD**, Dynavax: Grant/Research Support|GlaxoSmithKline: Grant/Research Support|Moderna: Grant/Research Support|Pfizer: Grant/Research Support **Vennis Hong, MPH**, Pfizer: Grant/Research Support **Sally Shaw, DrPH, MPH**, Pfizer: Grant/Research Support **Jeff Slezak, MS**, Dynavax Technologies: Grant/Research Support|Pfizer: Grant/Research Support **Julie Stern, MPH**, GlaxoSmithKline: Grant/Research Support|Pfizer: Grant/Research Support|Sanofi: Grant/Research Support **Hung Fu Tseng, PhD MPH**, GlaxoSmithKline: Grant/Research Support|Moderna: Grant/Research Support

